# The risk of metabolic syndrome as a result of lifestyle among Ellisras rural young adults

**DOI:** 10.1038/s41371-018-0076-8

**Published:** 2018-06-05

**Authors:** M. D. Sekgala, K. D. Monyeki, A. Mogale, Z. J. Mchiza, W. Parker, S. R. Choma, H. M. Makgopa

**Affiliations:** 1 Department of Physiology and Environmental Health, University of Limpopo, Polokwane, South Africa; 2 Population Health, Health System and Innovations, Human Science Research Council, Cape Town, South Africa; 3 Department of Biochemistry, Sefako Makgatho, Health Science University, Pretoria, South Africa; 4 Faculty of Community and Health Sciences, School of Public Health, University of the Western Cape, Bellville, South Africa; 5 Department of Pathology and Medical Sciences, University of Limpopo, Polokwane, South Africa

## Abstract

The study aimed to investigate the association between metabolic syndrome (MetS) and lifestyle risk factors among Ellisras adults. A cross-sectional study was conducted on 624 adults (306 males and 318 females). MetS was defined according to the criteria of the International Diabetes Federation. The prevalence of MetS was 23.1% (8.6% males and 36.8 % females). Females appeared to have higher mean values for waist circumference (WC), fasting blood glucose (FBG), total cholesterol (TCHOL) and low-density lipoprotein cholesterol (LDL-C), while males had high mean values for high-density lipoprotein cholesterol (HDL-C), triglycerides (TG), systolic blood pressure (SBP) and diastolic blood pressure (DBP). No significant age and gender differences were observed for dietary intake. Significantly more females (51.9%) presented with increased WC than males (4.6%). Participants who had a high dietary energy intake were significantly less likely to present with larger WC (OR: 0.250 95% CI [0.161; 0.389]), low HDL-C (OR: 0.306 95% CI [0.220; 0.425]) and high LDL-C (OR: 0.583 95% CI [0.418; 0.812]) but more likely to present with elevated FBG (OR: 1.01 95% CI [0.735; 1.386]), high TCHOL (OR: 1.039 95% CI [0.575; 1.337]), high TG (OR: 1.186 95% CI [0.695; 2.023]) and hypertension (OR: 5.205 95% CI [3.156; 8.585]). After adjusting for age, gender, smoking, and alcohol status, high energy intake was more than two times likely to predict MetS in adults with a large WC (OR: 2.766 95% CI [0.863; 3.477] and elevated FBG (OR: 2.227 95% CI [1.051; 3.328]). Therefore, identifying groups that are at an increased risk and those that are in their early stages of MetS will help improve and prevent the increase of the MetS in the future.

## Introduction

Metabolic syndrome (MetS) is a global problem associated with the clustering of several cardiovascular risk factors [1, 2]. Contrary to earlier thoughts, MetS is no longer rare in Africa, especially in medium-income countries such as South Africa [3]. South African evidence suggests an upsurge of non-communicable diseases (NCDs) amidst the existence of communicable diseases (CDs) such as HIV/AIDS and tuberculosis. Moreover, NCDs and CDs in the country are influenced by socio-demographic factors; and thus tend to be more prominent in certain segments of the population [1, 4–6]. The South African population is comprised of various ethnic groups [7]. Patterns of NCDs and CDs in this regard understandably vary by ethnicity, possibly due to ethnic and cultural differences. More importantly, the rise in NCDs in the country can be explained by the rapid nutrition transition associated with urbanization, the adoption of “Westernized” diets (diets high in fat, added sugar, and salt) and lifestyles (sedentary activity, excessive alcohol intake, and smoking) as well as the diversity of cultural and ethnic beliefs [8, 9].

Among others, the consequences of the aforementioned diet and lifestyle are insulin resistance, hyperinsulinaemia, central obesity, hypertension, elevated total cholesterol (TCHOL) and low-density lipoprotein cholesterol (LDL-C), dyslipidaemia (increase in plasma triglycerides [TG]) and a decrease in high-density lipoprotein cholesterol (HDL-C). Substantial evidence suggests that these consequences are components of MetS [1, 2]. Furthermore, there is evidence that emphasizes obesity as the main component of MetS [1, 2, 10]. Obesity is defined as the accumulation of excess body fat, which manifests as increased weight, or the centralization of body fat (indicated as higher than normal waist circumference (WC)) [1]. In Africa, different measures of body fat accumulation have been used as primary indicators for MetS. For instance, some studies emphasize body mass index (BMI), while others emphasize WC or waist-to-hip ratio (WHR) as the standard measures for MetS. In Cameroon, researchers used WHR (WHR>0.9 (males) and >0.85 (females) to measure body fat centralization in rural dwellers, while they used WC (as defined by both National Cholesterol Education Program (NCEP) and International Diabetes Federation (IDF)) in urban dwellers [10]. In South Africa, WC (≥86 cm for men and ≥92 cm for women) has been used as a predictor of the presence of at least two other components of MetS in a rural South African black community in KwaZulu-Natal [4]. In this study, it was also shown that MetS was more prevalent in women than in men. In fact, in South Africa metabolic diseases are expected to increase dramatically in women due to the rising rates of obesity and dyslipidaemia in this gender [11]. Body fat centralization was also defined according to the World Health Organization (WHO) classification as WC (≥102 cm and ≥88 cm for males and females, respectively) in Benin, while BMI was used in Gambia (≥30 kg/m^2^) and Nigeria (≥25–29.9 kg/m^2^ and ≥30 kg/m^2^) [12, 13]. This therefore poses significant challenges for country comparisons of the extent of MetS on the African continent. However, it is important to be cognisant of the pivotal role that the accumulation of body fat, particularly central obesity, plays in the aetiology of MetS in all these African studies [4, 10–13].

Moreover, it is important to be mindful of the other risk factors for MetS, namely, unhealthy diets and risky lifestyles [1, 2]. For example, the current diet consumed by South Africans needs close monitoring as it is thought to fuel the prevalence of MetS in the country [14]. In fact, the consumption of high-fat and high-sugar diets, especially by those who have financial constraints is undesirable. Most South African households cannot afford a healthy diet and therefore rely on a diet that is, in most cases, energy dense (high in added sugar and total fat) and high in salt and saturated fat, while it is deficient in nutrients [15]. This diet therefore influences the blood biochemical profile of the individuals who consume it, thereby subjecting them to metabolic diseases.

The study aimed to investigate the association between MetS and lifestyle risk factors among young rural South African adults aged 18–30 years. This age group was selected since young adults in rural South Africa have been identified as having a high risk of being undiagnosed for NCDs [16, 17]. Emphasis was placed on differentiating the risk of MetS by age and gender. The outcomes of the current study therefore will contribute to MetS prevention efforts, especially in vulnerable populations, namely, rural and young South Africans [4, 17].

## Methods

### Geographical area

Ellisras (known as Lephalale) is considered as one of the deep rural areas in the western part of the Limpopo province in South Africa. The villages are approximately 70 km away from the Ellisras town (23°40 S 27°44 W), adjacent to the Botswana border. The population is about 50,000 dispersed across 42 settlements [18]. The main sources of employment for the Ellisras residents are the Iscor coal mine and Matimba electricity power station. The remaining workforce mostly is involved in subsistence farming and cattle rearing, while a few are involved in education and civil services. Poverty, unemployment, and low life expectancy plays an important role in rural South African settings and the Ellisras rural population is not exempted from this [19].

### Sample and research design

A total of 624 adults (306 males and 318 females) aged 18–30 years who are part of the Ellisras Longitudinal Survey (ELS) participated in the current study [20, 21]. The Ethics Committee of the University of Limpopo granted ethical approval prior to the survey and the participants signed the informed consent forms.

### Data collection

#### Dietary intake

Dietary intake was measured using a validated 24 h recall method [22]. Senior Northern Sotho-speaking dietetic students of the University of Limpopo, specifically those trained to use the 24-h recall method, completed interviews with participants regarding their dietary intake over the previous 24 h. For each participant, interviews took place on one weekday and on one weekend day. An average of 2 days 24-h dietary intake was then made for each participant. Estimated portion sizes of foods consumed were recorded in as much detail as possible, using a pretested questionnaire and food models, simulating average portions of local foods [23]. A self‑administered questionnaire was used to collect data on lifestyle factors, including smoking and alcohol intake.

#### Anthropometric and blood pressure (BP) measurements

Anthropometric measurements (WC) were conducted on all study participants, according to standard procedures of the International Society for the Advancement of Kinanthropometry [24]. The WC measurements were taken to the nearest 0.1 cm, using a soft measuring tape.

Using an electronic Micronta Monitoring Kit, three BP readings were taken after the subject had been seated for 5 min or longer. The bladder of the device contains an electronic infrasonic transducer that monitors the BP and pulse rate, displaying these concurrently on the screen. This versatile instrument has been designed for research and clinical purposes [25].

#### Biochemical parameters

Fasting venous blood specimens were collected from all the participants for the measurement of fasting blood glucose (FBG), TCHOL, TG and HDL-C. Blood specimens for the measurement of fasting plasma glucose (FVPG) were drawn into fluoride tubes. The FVPG was measured using the glucose oxidase method, on a Beckman LX20^®^ auto-analyser (Beckman Coulter, Fullerton, CA) after the samples were centrifuged within 4 h. The enzymatic assay kits on a Beckman LX20^®^ auto-analyser (Beckman Coulter, Fullerton, CA) were used to measure serum lipid profile. High LDL-C was calculated using Friedewald equation [26]:

$${\mathrm{(}}\left[ {{\mathrm{LDL-C}}} \right] = \left[ {{\mathrm{TCHOL}}} \right]-\left[ {{\mathrm{HDL-C}}} \right]-\left[ {{\mathrm{triglyceride}}} \right]{\mathrm{/5)}}$$

### Criteria for MetS diagnosis

MetS was diagnosed using the new harmonized guidelines of the IDF, which requires large (WC) (≥94 cm males, ≥80 cm females plus two of the following criteria: reduced HDL-C (<1.0 mmol/L males; <1.3 mmol/L females), high TG (≥1.7 mmol/L), elevated BP (≥130 mm Hg systole and/or ≥85 mm Hg diastole), FBG (≥5.6 mmol/L) [27] and high LDL-C (≥3 mmol/L) [28].

### Statistical analysis

Descriptive statistics were used to describe the participant’s characteristics and data were presented using numbers, percentages, medians, means, and standard deviations. Linear regression was used to investigate the association between dietary intake and MetS risk factors. Dietary intake variables used in the linear regression method were log transformed prior to analysis because of their skewed distribution. Logistic regression analysis was used to investigate the influence of dietary intake on MetS risk factors. The statistical package of the social sciences (SPSS) version 23.0 was used for data analysis. A *p*-value of <0.05 was considered statistically significant. Dietary data were analysed using local food tables and software [29] and compared to the recommended intake [30].

## Results

Table 1 shows the mean values for the MetS risk factors and dietary intake of the participants. Overall, females appear to have higher mean values for WC, FBG, TCHOL and LDL-C, while males have high mean values for HDL-C, TG, SBP and DBP. This trend is consistent, when disaggregating the sample by age groups. However, the only significant differences between males and females were recorded for WC (75.09 ± 9.53 and 82.14 ± 14.37, respectively) and SBP (125.91 ± 12.78 and 114.23 ± 10.84, respectively), with this significant gender difference also visible in the 25–30-year-old group. With regard to differences between age groups, most risk factors were more prevalence in the older age group (25–30 years), except for FBG and HDL-C (5.56 ± 0.91 and 1.16 ± 0.31, respectively) that were higher in the 18–24-year-old group compared to 1.16 ± 0.31) and 1.14 ± 0.35, respectively) in the 25–30-year-old group). There were no significant differences between age groups for all the measured risk factors except for the DBP where the mean value was 68.78 ± 9.37) in the 18–24-year-old group compared to 70.96 ± 10.05 in the 25–30-year-old group (*p* < 0.05). While no significant differences were observed for dietary intake between males and females, the largest difference observed between males and females were for energy intake, where females had a higher median energy intake than males [3474 (3482.00) vs 3029.0 (3874.00), respectively]. This relationship was different when the data was disaggregated by age in that, in the 18–24-year-old age group, males reported a higher energy intake [3520.00 (3646.50)] than females [3314.00 (2919.00)], whereas females reported a higher energy intake [3674.00 (3992.50)] than males [2886.00 (3967.00)] in the 25–30-year-old group. With regard to macronutrients, females consumed more carbohydrates, added sugar, fibre and saturated fat; while males consumed more total fats, proteins, monounsaturated and polyunsaturated fats.

**Table 1:** Descriptive statistic for metabolic syndrome risk factors of Ellisras adults by age group and gender

Risk factors	18–24 years			25–30 years			18–30 years		
	Males (*n* = 103)	Females (*n* = 101)	Total (*N* = 204)	Males (*n* = 203)	Females (*n* = 217)	Total (*N* = 420)	Males (*n* = 306)	Females (*n* = 318)	Total (*N* = 624)
	Mean ± SD	Mean ± SD	Mean ± SD	Mean ± SD	Mean ± SD	Mean ± SD	Mean ± SD	Mean ± SD	Mean ± SD
WC (cm)	72.07 ± 7.27	78.05 ± 13.36	75.03 ± 11.11	76.62* ± 10.17	84.04* ± 14.46	80.46 ± 13.09	75.09* ± 9.53	82.14* ± 14.37	78.68 ± 12.73
FBG (mmol/L)	5.54 ± 0.91	5.78 ± 0.91	5.56 ± 0.91	5.40 ± 0.84	5.63 ± 1.77	1.16 ± 0.31	5.45 ± 0.87	5.62 ± 1.55	5.53 ± 1.26
TC (mmol/L)	4.02 ± 0.87	4.07 ± 1.03	4.04 ± 0.95	4.04 ± 0.95	4.35 ± 1.13	4.20 ± 1.06	4.03 ± 0.92	4.62 ± 1.11	4.15 ± 1.03
HDL-C (mmol/L)	1.23 ± 0.34	1.09 ± 0.28	1.16 ± 0.31	1.19 ± 0.39	1.10 ± 0.31	1.14 ± 0.35	1.20 ± 0.37	1.10 ± 0.30	1.15 ± 0.34
TG (mmol/L)	0.96 ± 0.60	0.87 ± 0.48	0.92 ± 0.54	1.11 ± 0.67	1.00 ± 0.52	1.05 ± 0.60	1.06 ± 0.65	0.96 ± 0.51	1.01 ± 0.59
LDL-C (mmol/L)	2.61 ± 0.71	2.80 ± 0.89	2.71 ± 0.81	2.63 ± 0.81	3.05 ± 0.96	2.85 ± 0.92	2.62 ± 0.78	2.97 ± 0.95	2.80 ± 0.89
SBP (mm Hg)	123.20 ± 12.30	112.61 ± 9.16	117.95 ± 12.06	127.29* ± 12.37	114.98* ± 11.49	120.93 ± 13.41	125.91** ± 12.78	114.23** ± 10.84	119.96 ± 13.05
DBP (mm Hg)	68.89 ± 9.58	68.66 ± 9.20	68.78* ± 9.37	72.73 ± 10.35	69.31 ± 9.50	70.96* ± 10.05	71.44 ± 10.34	69.10 ± 9.39	70.25 ± 9.88
**Dietary intake**	**Median (IQR)**	**Median (IQR)**	**Median (IQR)**	**Median (IQR)**	**Median (IQR)**	**Median (IQR)**	**Median (IQR)**	**Median (IQR)**	**Median (IQR)**
Energy (kj)	3520.00 (3646.50)	3314.00 (2919.00)	3486.00 (3299.50)	2886.00 (3967.50)	3674.00 (3992.50)	3213.50 (3953.50)	3029.0 (3874.00)	3474 (3482.00)	3310.0 (3591.00)
Fatty acids (%)	23.18 (17.73)	20.71 (22.37)	21.43 (20.01)	22.22 (26.10)	22.75 (25.80)	22.50 (26.80)	22.6 (21.51)	22.1 (24.26)	22.3 (23.50)
Protein (%)	14.48 (11.67)	11.55 (9.24)	13.17 (10.45)	12.07 (13.75)	11.68 (12.78)	12.03 (13.36)	12.9 (12.34)	11.7 (11.19)	12.3 (11.76)
Carbohydrate (%)	61.81 (24.49)	65.98 (28.80)	63.31 (23.73)	63.76 (35.57)	62.29 (33.42)	62.79 (34.60)	62.8 (30.82)	63.7 (30.91)	63.0 (31.41)
Added sugar (g)	24.40 (39.83)	34.70 (49.30)	27.70 (40.65)	24.00 (49.70)	25.80 (38.45)	25.80 (39.90)	24.0 (45.50)	26.0 (36.00)	25.8 (39.50)
Fibre (g)	5.60 (8.18)	5.90 (7.80)	5.70 (7.85)	3.80 (7.15)	4.80 (9.85)	4.05 (8.43)	4.3 (7.00)	5.1 (9.00)	4.6 (8.40)
SFAs (%)	5.83 (6.42)	4.56 (8.42)	5.28 (7.75)	4.14 (10.06)	5.28 (10.62)	5.02 (10.44)	4.8 (8.54)	5.0 (9.91)	4.9 (9.36)
MUFAs (%)	8.20 (9.55)	5.39 (10.50)	6.67 (10.03)	5.19 (13.77)	6.97 (14.95)	6.48 (14.79)	6.6 (11.75)	6.4 (14.01)	6.5 (12.90)
PUFAs (%)	5.07 (7.37)	2.97 (7.67)	4.02 (7.63)	2.97 (7.74)	4.16 (8.61)	3.44 (8.39)	3.7 (7.57)	3.4 (8.18)	3.7 (7.95)
Trans fatty acids (%)	0.18 (1.10)	0.12 (1.05)	0.14 (1.05)	0.12 (0.40)	0.12 (1.27)	0.12 (0.81)	0.1 (0.49)	0.1 (1.17)	0.1 (0.95)

Data on lipid profile and anthropometry are presented as mean ± SD, while dietary intake data are presented as median (IQR)

*n* number of participants, *WC* waist circumference, *FBG* fasting blood glucose, *TC* total cholesterol, *HDL* high-density lipoprotein, *LDL* low-density lipoprotein, *TG* triglycerides, *CHO* carbohydrates, *SBP* systolic blood pressure, *DBP* diastolic blood pressure, *SFA* saturated fatty acid, *MUFA* monounsaturated fatty acid, *PUFA* polyunsaturated fatty acid, *TFA* trans fatty acid, *IQR* interquartile range **p < 0.001; *p < 0.05

**Fig. 1:**
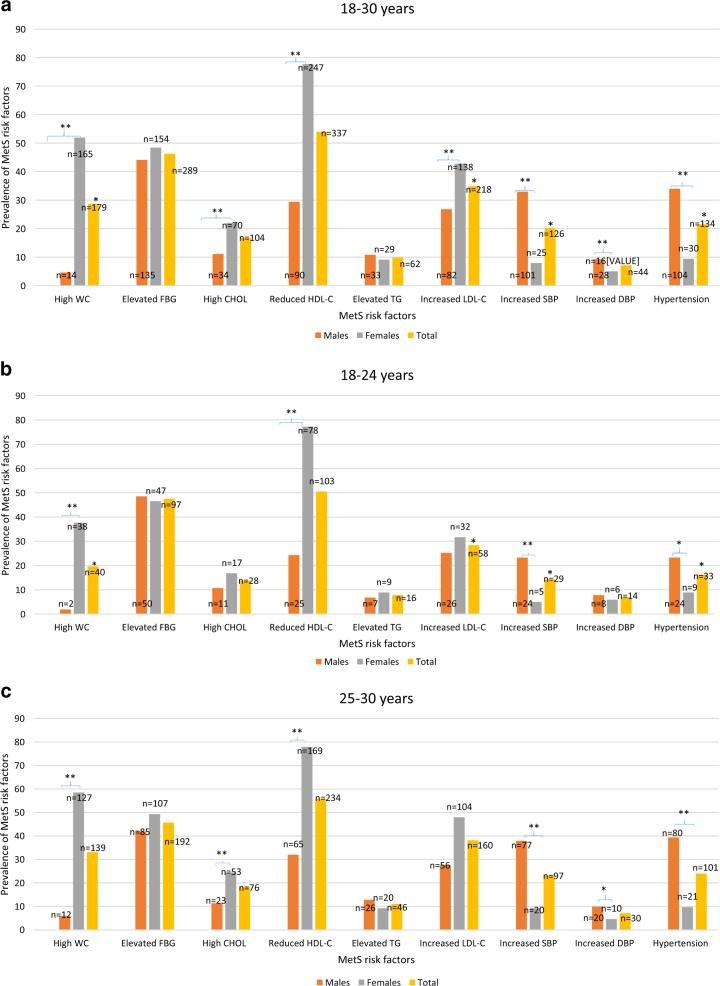
**a** Prevalence of the metabolic syndrome risk factors in the total sample, males and females in the age group 18–30 years of Ellisras young adults. **b** Prevalence of metabolic syndrome risk factors in the total sample, males and females in the age group 18–24 years of Ellisras young adults. **c** Prevalence of metabolic syndrome risk factors in the total sample, males and females in the age group 25–30 years of Ellisras young adults

Figure 1a shows the observed prevalence of each MetS risk factor of the total sample of participants. Overall, significantly more females presented with increased WC than males (51.9 vs 4.6%). The trend of gender differences was also observed for elevated TCHOL (22.0% for females vs 11.1% for males) and LDL-C (42.8% for females vs 26.8% for males), as well as the reduced HDL-C (77.7% for females vs 29.4% for males). Conversely, significantly more males presented with increased SBP (*p* < 0.001), DBP (*p* < 0.05) and hypertension (*p* < 0.001), (33.0, 9.2 and 34.0%, respectively) when compared to their female counterparts (7.9, 5.0 and 9.4%, respectively). Overall, the risk factors with the highest prevalence were reduced HDL-C, elevated FBG and increased LDL-C (ranging from 54.0, 46.3 and 34.9%, respectively). The risk factors with the lowest prevalence were increased DBP, increased TG and high TCHOL (ranging from 7.1, 9.9 and 16.7%, respectively).

The gender difference trend was observed even when the sample data was disaggregated by age groups (Fig. 1b) in that significantly more females aged 18–24 years presented with larger WC and reduced HDL (37.6 and 77.2%, respectively) than males (1.9 and 24.3%, respectively). Males in the 18–24-year-old group had significantly high DBP and hypertension than females (23.3 vs 5.0% and 23.3 vs 8.9%, respectively). Overall, the risk factors with the highest prevalence in this particular age group were reduced HDL-C, elevated FBG and increased LDL-C (ranging from 50.5, 47.5 and 28.4%, respectively). The risk factors with the lowest prevalence were increased DBP, elevated TG and high TCHOL (ranging from 6.9, 7.8 and 13.7%, respectively).

Consistency trends were observed in the 25–30-year-old group (Fig. 1c), as large WC and reduced HDL-C were significantly higher in more females than males (58.5 vs 5.9% and 77.9 vs 32.0%, respectively). In addition, significantly more females (24.4%) had elevated TCHOL than males (11.3%). Similarly, as observed in the 18–24-year-old group, more males than females presented with high SBP (37.9 vs 9.2%), DBP (9.9 vs 4.6%) and hypertension (39.4 vs 9.7%), respectively). Overall, the risk factors with the highest prevalence in the 25–30-year-old group were reduced HDL-C, elevated FBG and increased LDL-C (ranging from 55.7, 45.7 and 38.1%, respectively). The lowest prevalence risk factors are increased DBP, elevated TG and high TCHOL (ranging from 7.1, 11.0 and 18.1%, respectively).

With regard to dietary intake, overall, significantly more males than females reported a high total energy (95.8 vs 17.6%), high protein (5.9 vs 1.6%) and low fibre intake (99.3 vs 96.2%). This trend remained significant for a high total energy when data were disaggregated by age group, where 99.0% of males within the 18–24-year-old group had high total energy intake compared to 7.9% females and 94.1% of males within the 25–30-year-old group had high total energy intake compared to 22.1% females. However, the trend for a high protein and low fibre intake only remained significant in the 25–30-year-old group where and 7.4 and 99.5% of males reported a high protein and low fibre intake compared to 1.8 and 95.9% of females, respectively.

On the other hand, as shown in Table 2, more females reported a high added sugar intake compared to males (56.9 vs 48.4%). This remained significant in the 18–24-year-old group where 66.3% of females reported a high prevalence of added sugar compared to 48.5% of males. In the 25–30-year-old group, significantly more females (27.6%) reported intakes of high trans fatty acid diets compared to males (17.7%).

**Table 2:** The prevalence of dietary intake of the participants

Dietary intake	18–24 years			25–30 years			18–30 years		
	Males (*n* = 103)	Females (*n* = 101)	Total (*N* = 204)	Males (*n* = 203)	Females (*n* = 217)	Total (*N* = 420)	Males (*n* = 306)	Females (*n* = 318)	Total (*N* = 624)
	(%) *N*	(%) *N*	(%) *N*	(%) *N*	(%) *N*	(%) *N*	(%) *N*	(%) *N*	(%) *N*
High energy male >10,626; female >8465	99.0** (102)	7.9** (8)	53.9 (110)	94.1** (191)	22.1** (48)	56.9 (239)	95.8** (293)	17.6** (56)	55.9 (349)
High fatty acids ≥35%	18.4 (19)	19.8 (20)	19.1 (39)	23.6 (48)	24.0 (52)	23.8 (100)	21.9 (67)	22.6 (72)	22.3 (139)
High protein ≥35%	2.9 (3)	1.0 (1)	2.0* (4)	7.4* (15)	1.8* (4)	4.5* (19)	5.9* (18)	1.6* (5)	3.7 (23)
High carbohydrate ≥65%	40.8 (42)	50.5 (51)	45.6 (93)	48.3 (98)	44.7 (97)	46.4 (195)	45.8 (140)	46.5 (148)	46.2 (288)
High added sugar <25 g	48.5* (50)	66.3* (67)	57.4 (117)	48.3 (98)	52.5 (114)	50.5 (212)	48.4* (148)	56.9* (181)	52.7 (329)
Low fibre male = 38 g; female = 25 g	99.0 (102)	97.0 (98)	98.0 (200)	99.5* (202)	95.9* (208)	97.6 (410)	99.3* (304)	96.2* (306)	97.8 (610)
High saturated fatty acids <10%	22.3 (23)	23.8 (24)	23.0 (47)	27.6 (56)	30.9 (67)	29.3 (123)	25.8 (79)	28.6 (91)	27.2 (170)
High monounsaturated fatty acids ≥20%	4.9 (5)	6.9 (7)	5.9 (12)	7.4 (15)	7.4 (16)	7.4 (31)	6.6 (20)	7.2 (23)	6.9 (43)
High polyunsaturated fatty acids ≥10%	22.3 (23)	19.8 (20)	21.1 (43)	18.2 (37)	22.6 (49)	20.5 (86)	19.6 (60)	21.7 (69)	20.7 (129)
High trans fatty acids <1%	26.2 (27)	25.7 (26)	26.0 (53)	17.7* (36)	27.6* (60)	22.9 (96)	20.6 (63)	27.0 (86)	23.9 (149)

*n* number of participants **p < 0.001; *p<0.05

With regard to age groups, the only significant difference observed was for reported intake of high protein, where more people in the 25–30-year-old group (4.5%) reported a high protein intake than those in the 18–24-year old group (2.0%).

It is important to note that the details of the outcomes for MetS are presented elsewhere [31]. A snapshot of these outcomes are as follows: Overall, the total prevalence of MetS was 23.1% (8.6% males and 36.8% females). When the sample data was disaggregated by age groups, the prevalence of MetS in the 18–24-year-old group was 20.1% (9.8% males and 30.8% females) and 25.0% (7.7% males and 30.8% females) in the 25–30-year-old group.

Table 3 shows the linear regression analysis undertaken to show the association of each log dietary intake variables with different MetS risk factors. The results showed no association between log total energy, log added sugar, log saturated fatty acid (SFA) and log monounsaturated fatty acid (MUFA) with metabolic risk factors. There was a low and negative significant association between log fibre with SBP and DBP (*β*:−0.004, *p* = 0.003 and *β*:−0.004, *p* = 0.046), respectively. After adjusting for potential confounding factors, log fibre was also associated with FBG (*β*:−0.028, *p* = 0.046). Log polyunsaturated fatty acids (PUFAs) was inversely associated with FBG, HDL-C and SBP crude. Log trans fatty acids was inversely associated with WC, HDL-C and SBP crude. Both log PUFAs and log trans fatty acids were not associated with any metabolic risk factors after adjusting for potential confounding factors. Log protein was inversely associated with SBP both crude and adjusted for potential confounding factors.

**Table 3:** Regression coefficient, 95% CI and *p*-value in the association of dietary intake with various metS risk factors of Ellisras adults

	Energy (kj)			Added sugar (g)			Fibre (g)			Saturated fatty acids (%)			PUFAs (%)			MUFAs (%)			Trans fatty acids (%)			Carbohydrates (%)			Protein (%)			Fatty acids (%)		
	*β*	SE	*p*-Value	*β*	SE	*p*-Value	*β*	SE	*p*-Value	*β*	SE	*p*-Value	*β*	SE	*p*-Value	*β*	SE	*p*-Value	*β*	SE	*p*-Value	*β*	SE	*p*-Value	*β*	SE	*p*-Value	*β*	SE	*p*-Value
Crude																														
Age (years)	−0.034	0.009	0	−0.016	0.014	0.257	−38	0.009	0	−0.02	0.009	0.036	−0.029	0.009	0.002	−0.026	0.01	0.013	−0.008	0.006	0.18	−0.033	0.01	0.001	−0.065	0.016	0	−0.069	0.02	0
Gender	0.009	0.036	0.798	0.108	0.055	0.052	0.038	0.035	0.288	0.004	0.038	0.909	−0.004	0.037	0.905	−0.005	0.042	0.9	0.033	0.025	0.189	0.007	0.039	0.851	−0.04	0.064	0.535	−0.008	0.07	0.918
WC (cm)	−0.002	0.001	0.131	0.001	0.002	0.864	−0.001	0.001	0.433	−0.001	0.001	0.33	−0.002	0.001	0.131	−0.002	0.002	0.144	−0.002	0.001	0.047	0.001	0.002	0.823	−0.002	0.003	0.333	−0.004	0	0.169
FBG (mmol/L)	−0.02	0.014	0.149	0.013	0.022	0.567	−0.03	0.014	0.034	−0.007	0.015	0.644	−0.016	0.016	0.043	−0.012	0.017	0.471	−0.005	0.01	0.594	−0.016	0.015	0.298	−0.025	0.025	0.332	−0.031	0.03	0.279
TC (mmol/L)	−0.011	0.017	0.538	−0.008	0.027	0.501	−0.015	0.017	0.371	−0.016	0.018	0.379	−0.007	0.018	0.688	−0.016	0.02	0.435	−0.001	0.012	0.911	−0.031	0.019	0.099	−0.034	0.031	0.275	−0.046	0.04	0.199
HDL-C (mmol/L)	0.027	0.053	0.612	−0.097	0.082	0.237	−0.004	0.052	0.444	0.071	0.056	0.203	0.042	0.055	0.44	0.067	0.062	0.278	0.074	0.037	0.045	−0.025	0.057	0.668	−0.024	0.094	0.801	−0.004	0.11	0.971
TG (mmol/L)	−0.033	0.031	0.274	−0.027	0.047	0.572	−0.026	0.03	0.399	−0.032	0.032	0.317	−0.024	0.032	0.453	−0.022	0.036	0.529	−0.03	0.021	0.159	−0.015	0.033	0.65	−0.043	0.055	0.427	−0.058	0.06	0.356
LDL-C (mmol/L)	−0.014	0.02	0.482	0.006	0.031	0.848	−0.006	0.021	0.79	−0.028	0.021	0.189	−0.014	0.021	0.52	−0.028	0.024	0.237	−0.009	0.014	0.51	−0.036	0.022	0.105	−0.037	0.036	0.311	−0.054	0.04	0.191
SBP (mm Hg)	−0.002	0.001	0.096	0.002	0.001	0.11	−0.004	0.001	0.003	−0.002	0.001	0.189	−0.003	0.001	0.05	−0.002	0.002	0.159	−0.002	0.001	0.019	−0.002	0.001	0.118	−0.006	0.002	0.022	−0.006	0	0.039
DBP (mm Hg)	−0.002	0.002	0.246	0.001	0.003	0.94	−0.004	0.002	0.046	−0.002	0.002	0.287	−0.003	0.002	0.132	−0.003	0.002	0.183	−0.002	0.001	0.163	−0.004	0.002	0.051	−0.007	0.003	0.039	−0.007	0	0.074
Adjusted for age, gender and energy																														
Age (years)	−0.031	0.009	0.001	−0.015	0.014	0.3	−0.037	0.009	0	−0.016	0.01	0.097	−0.026	0.01	0.008	−0.002	0.011	0.615	−0.004	0.006	0.525	−0.032	0.01	0.002	−0.062	0.016	0	−0.063	0.02	0.001
Gender	0.001	0.045	0.988	0.165	0.07	0.019	−0.002	0.044	0.956	0.003	0.048	0.956	−0.002	0.047	0.64	−0.002	0.053	0.964	0.036	0.031	0.25	−0.002	0.049	0.96	−0.199	0.08	0.137	−0.049	0.09	0.59
WC (cm)	0.001	0.002	0.788	−0.002	0.002	0.419	0.001	0.002	0.594	0.001	0.002	0.931	0.001	0.002	0.795	−0.001	0.002	0.713	−0.001	0.001	0.183	0.002	0.002	0.328	0.002	0.003	0.441	0.001	0	0.932
FBG (mmol/L)	−0.017	0.014	0.23	0.008	0.022	0.708	−0.028	0.014	0.046	−0.002	0.015	0.887	−0.011	0.015	0.466	−0.006	0.017	0.715	−0.001	0.01	0.911	−0.013	0.016	0.387	−0.017	0.025	0.501	−0.023	0.03	0.436
TC (mmol/L)	−0.198	0.302	0.512	−0.436	0.471	0.355	0.017	0.295	0.953	−0.217	0.321	0.5	−0.009	0.315	0.977	−0.203	0.354	0.567	−0.124	0.21	0.557	0.245	0.329	0.455	−0.251	0.536	0.64	−0.226	0.62	0.714
HDL-C (mmol/L)	0.223	0.304	0.465	0.35	0.475	0.462	−0.049	0.297	0.868	0.305	0.324	0.346	0.054	0.315	0.865	0.28	0.357	0.433	0.21	0.212	0.322	0.239	0.331	0.47	0.233	0.54	0.666	0.236	0.62	0.705
TG (mmol/L)	0.03	0.069	0.664	0.081	0.107	0.45	−0.001	0.067	0.991	0.034	0.073	0.646	0.002	0.072	0.982	0.047	0.081	0.556	0.014	0.048	0.776	0.064	0.075	0.392	0.049	0.122	0.687	0.046	0.14	0.744
LDL-C (mmol/L)	0.194	0.304	0.524	0.431	0.475	0.364	−0.021	0.297	0.944	0.19	0.324	0.558	0.003	0.318	0.991	0.179	0.357	0.615	0.113	0.212	0.594	0.214	0.331	0.517	0.238	0.54	0.659	0.193	0.62	0.756
SBP (mm Hg)	−0.002	0.002	0.278	0.004	0.003	0.176	−0.004	0.002	0.051	−0.002	0.002	0.365	−0.003	0.318	0.174	−0.002	0.002	0.98	−0.002	0.001	0.213	−0.001	0.002	0.594	−0.007	0.004	0.051	−0.006	0	0.161
DBP (mm Hg)	0.001	0.002	0.662	−0.002	0.004	0.634	0.001	0.002	0.641	0.001	0.003	0.933	0.001	0.003	0.881	0.001	3E−04	0.895	0.001	0.002	0.852	−0.002	0.003	0.469	0.001	0.004	0.92	0.001	0.01	0.929

*WC* waist circumference, *FBG* fasting blood glucose, *TC* total cholesterol, *HDL* high density lipoprotein, *LDL* low-density lipoprotein, *TG* triglycerides, *CHO* carbohydrates, *SBP* systolic blood pressure, *DBP* diastolic blood pressure, *SFA* saturated fatty acid, *PUFA* polyunsaturated fatty acid, *MUFA* monounsaturated fatty acid, *TFA* trans fatty acid

Table 4 shows that participants who had high dietary energy intake were significantly less likely to present with larger WC (odds ratio (OR): 0.250 95% confidence interval (CI) [0.161; 0.389]), low HDL-C (OR: 0.306 95% CI [0.220; 0.425]) and high LDL-C (OR: 0.583 95% CI [0.418; 0.812]) but more likely to present with elevated FBG (OR: 1.01 95% CI [0.735; 1.386]), high TCHOL (OR: 1.039 95% CI [0.575; 1.337]), high TG (OR: 1.186 95% CI [0.695; 2.023] and hypertension (OR: 5.205 95% CI [3.156; 8.585]) crude. After adjusting for age, gender, smoking and alcohol status, high energy intake was more than two times more likely to predict MetS in adults with a large WC (OR: 2.766 95% CI [0.863; 3.477] and elevated FBG (OR: 2.227 95% CI [1.051; 3.328]). Furthermore, low dietary fibre intake was nearly four time more likely to increase low HDL-C (OR: 3.864 95% CI [1.067; 13.988]) crude.

**Table 4:** Binary logistic regression analysis to show dietary predictors of metabolic syndrome risk factors in young adults (18–30 years) of Ellisras

	Energy (kj)			Added sugar (g)			Fibre (g)			Saturated fatty acids (%)			Trans fatty acids (%)		
	OR	95% CI	*p*-Value	OR	95% CI	*p*-Value	OR	95% CI	*p*-Value	OR	95% CI	*p*-Value	OR	95% CI	*p*-Value
Crude															
WC: male ≥102 cm, female ≥88 cm	0.25	(0.161; 0.389)	<0.001	1.005	(0.669; 1.509)	0.982	0.741	(0.164; 3.358)	0.741	1.052	(0.669; 1.654)	0.826	0.93	(0.574; 1.506)	0.767
FBG ≥5.6 mmol/L	1.01	(0.735; 1.386)	0.053	0.701	(0.512; 0.964)	0.029	0.638	(0.211; 1.925)	0.425	1.076	(0.756; 1.532)	0.683	1.424	(0.985; 2.060)	0.052
TC ≥5.1 mmol/L	1.039	(0.575; 1.337)	0.009	1.088	(0.714; 1.659)	0.693	0.83	(0.183; 3.765)	0.809	0.87	(0.537; 1.412)	0.574	1.215	(0.753; 1.961)	0.425
HDL-C: men <1 mmol/L, female <1.2 mmol/L	0.306	(0.220; 0.425)	<0.001	0.686	(0.500; 0.941)	0.019	3.864	(1.067; 13.988)	0.039	0.881	(0.619; 1.254)	0.482	0.739	(0.510; 1.070)	0.109
TG ≥1.7 mmol/L	1.186	(0.695; 2.023)	0.0531	0.846	(0.499; 1.436)	0.536	1.528	(0.334; 6.989)	0.585	0.838	(0.455; 1.542)	0.57	0.663	(0.336; 1.307)	0.235
LDL-C >3 mmol/L	0.583	(0.418; 0.812)	0.001	0.866	(0.623; 1.206)	0.395	0.74	(0.229; 2.388)	0.615	1.176	(0.816; 1.696)	0.386	0.54	(0.321; 0.906)	0.02
Hypertension ≥130/≥85 mm Hg	5.205	(3.156; 8.585)	<0.001	1.2424	(0.840; 1.836)	0.278	0.97	(0.941; 1.001)	0.054	0.716	(0.451; 1.138)	0.158	1.255	(0.858; 1.835)	0.242
Adjusted (age, gender, smoking and alcohol status)															
WC: male ≥102 cm, female ≥88 cm	2.766	(0.863; 3.477)	0.022	1.014	(0.614; 1.675)	0.957	0.401	(0.084; 1.903)	0.25	0.919	(0.508; 1.664)	0.78	1.143	(0.618; 2.115)	0.669
FBG ≥5.6 mmol/L	2.227	(1.051; 3.328)	0.033	0.706	(0.504; 0.988)	0.042	0.641	(0.208; 1.976)	0.439	1.027	(0.689; 1.530)	0.897	0.672	(0.441; 1.023)	0.053
TC ≥5.1 mmol/L	1.145	(0.556; 2.358)	0.714	1.2	(0.756; 1.903)	0.44	0.803	(0.171; 3.769)	0.781	1.394	(0.788; 2.467)	0.254	0.68	(0.385; 1.203)	0.185
HDL-C: men <1 mmol/L, female <1.2 mmol/L	1	(0.988; 1.000)	0.003	1.008	(1.003; 1.013)	0.002	1.046	(1.015; 1.157)	0.004	0.993	(0.966; 1.020)	0.601	1.022	(0.948; 1.103)	0.568
TG ≥1.7 mmol/L)	0.826	(0.316; 2.163)	0.698	0.772	(0.441; 1.351)	0.365	1.681	(0.357; 7.929)	0.511	1.127	(0.575; 2.211)	0.727	1.405	(0.666; 2.964)	0.372
LDL-C >3 mmol/L	1.191	(0.661; 2.145)	0.501	0.963	(0.670; 1.384)	0.863	0.638	(0.192; 2.116)	0.462	0.914	(0.596; 1.402)	0.68	0.87	(0.555; 1.363)	0.543
Hypertension ≥130/≥85 mm Hg	1.376	(0.618; 3.065)	0.434	0.95	(0.615; 1.468)	0.818	0.985	(0.953; 1.017)	0.35	1.131	(0.661; 1.936)	0.653	1.505	(0.824; 2.748)	0.183

*WC* increased waist circumference, *FBG* elevated fasting blood glucose, *TC* high total cholesterol, *HDL* low high-density lipoprotein, *LDL* high low-density lipoprotein, *TG* elevated triglycerides, *SBP* increased systolic blood pressure, *DBP* increased diastolic blood pressure, *OR* odds ratio

Those participants who consumed high trans fats were more likely to present with high FBG (OR:1.424 95% CI [0.985; 2.060]) but less likely to present with high LDL-C (OR: 0.540 95% CI [0.321; 0.906]) crude. However, after adding potential confounding factors, participants with high fatty acid were less likely to present with high FBG (OR: 0.672 95% CI [0.441; 1.023]). The rest of the dietary factors (protein, carbohydrates, PUFAs and MUFAs) were not included in the OR model since they did not meet the categorical data standard.

In summary, it seems as though high total dietary energy, high added sugar intake, low fibre, high SFA and trans fatty acids increased the likelihood of participants presenting with high WC, FBG, TCHOL, HDL-C, TG, LDL-C and hypertension.

## Discussion

Globally, the prevalence of the MetS is on the rise. Developing countries in Africa, such as South Africa, is not exempt from this. Interestingly, when the criteria for the Joint Statement definition of MetS is applied in South Africa, the MetS prevalence appears to be differentiated by ethnicity in that it is higher in black ethnic groups (>60%) compared to their white (±55%) counterparts [32]. However, when the criteria for IDF is applied, the prevalence is lower in black ethnic groups (46.5%) compared to their white counterparts (74.1%) [33]. Motala et al. (2011) [4] on the other hand showed that the MetS prevalence differs between genders in that more females (25, 21.2 and 16.8%) present with MetS when compared to their male counterparts (10, 11.2 and 7.9%) when applying the criteria for the JIS, IDF and ATP 111 definitions, respectively. Furthermore, when the criteria for the WHO definition is applied it appears as though the prevalence of MetS is estimated to be high (59.1%) in African countries such as Nigeria when compared to other developing countries like Turkey (19%) [34, 35]. However, when the NCEP-ATP III and IDF definition criteria are used, the MetS prevalence is shown to be higher in Turkey (38 and 42%, respectively) than in Africa (i.e. in Cameroon, ±1% and ±11%, respectively) [35, 36]. This therefore shows that the differences in the MetS prevalence observed in developing countries can be explained by the definition for MetS that is applied, as well as the gender and ethnicity of the population being studied [37–39].

The current study population prevalence of MetS was estimated using the IDF criteria, and overall, the prevalence of MetS was 23.1% (8.6% males and 36.8 % females) [31]. This prevalence is lower than the prevalence shown in South African coloured participants in the Erasmus et al. (2011) [33] study, but higher than the prevalence shown in black (9.5% for females and 6.8% for males) North West residents participating in the Hoebel et al. (2011) [32] study. Additionally, overall participants in the current study had a higher MetS prevalence than that reported in low-income black South Africans in the study by Owolabi et al. [40]. However, males in the current study had a lower MetS prevalence than males in Owolabi et al’s study. We have to bear in mind that the definition criteria used for MetS in these studies were the IDF criteria. On examining factors that seemed to influence MetS prevalence in the current study, it appears as though age and gender were the main determinants of this condition. In fact, being older influenced the health status of the participants especially the mean SBP and adiposity as shown by the values that were higher in the 25–30-year-old group than in the 18–24-year-old group. The majority of females also presented with larger WC and higher levels of TCHOL and lower levels of HDL-C. The results of the current study are somewhat corroborated by South African evidence that suggested that in the North West province that is closer to Ellisras (Limpopo province) had the majority of females (43.5%) with WC >88 cm when compared to 8% of males who present with WC that are >102 cm [41]. In the same survey, the mean SBP values also significantly differed by gender, with males presenting with higher mean values than females. SBP and DBP also seemed to increase with age. However, no significant gender differences were observed in terms of serum cholesterol.

Total energy intake in this study falls below the dietary reference intakes for both males and females. However, females tend to consume more energy than males. Consumption of excessive dietary energy have been shown in other studies conducted in black ethnic communities and other rural areas in South Africa [15]. With regard to the macronutrients consumed, females consumed more carbohydrates, added sugar, fibre, and saturated fat; while males consumed more total fats. These eating behaviours may be associated with the rapid nutrition transition in the country. What was striking in the current findings was that participants in the current study consumed less dietary fibre than the recommended dietary allowances of 38 g for males and 25 g for females of this age group. This outcome is in line with the outcomes of Wentzel-Viljoen et al. (2005) [42] where they showed that fibre intake is low in adults residing in the North West province in South Africa. This is a cause for concern given the increased benefits that people are likely to have if they consume this nutrient. In fact, substantiated evidence suggests that high dietary fibre intake lowers adiposity since it suppresses appetite [43]. Moreover, fibre is beneficial in that it expedites the movements of waste products through the intestinal tract, thereby decreasing the gut transit time to protect the gut from harmful waste that may support the development of different forms of gut cancers [44] Moreover, dietary fibre is important in that it has antidiabetic properties, inhibits the oxidation of LDL cholesterol, reduces platelet aggregation and later reduces ischaemic damage [45].

Literature on the association between MetS risk factors with dietary intakes is limited, particularly in the poor rural populations [46]. In the current study, dietary fibre was significantly associated with SBP and DBP. The same finding was reported by Moreno Franco et al. (2014) [47]. An intervention study showed that increased dietary fibre intake significantly reduced both DBP and SBP [48]. Further, there was an association between dietary fibre intake and FBG among the current study participants. These findings are consistent with the findings of Giacco et al. (2000) [49] in that high dietary intake improves the blood glucose level. The beneficial metabolic effects of dietary fibre intake included both an improvement in the daily blood glucose level and a reduction in the number of hypoglycaemic events [49]. PUFAs was significantly associated with FBP, HDL-C and SBP. Food rich in PUFA increases insulin sensitivity [50], glucose utilization and decreases insulin resistance and the risk of type 2 diabetes [51]. These results shows that PUFAs improves MetS risk factors. Protein was also associated with SBP in the current study. There is evidence that consumption of high protein has a protective effect against the MetS [52]. However, this association needs to be interpreted with caution given that consuming higher than recommended amounts of protein is associated with increased BP and hypertensive diseases [53] and is therefore still controversial in the management of MetS. In the current study, participants with a high energy intake were less likely to present with a large WC, low HDL-C and LDL-C. Similar results were reported by Bruscato et al. (2010) [54]. These results may be attributed to participants consuming less energy than recommended. These results have a policy implication in that they call upon urgent interventions including nutrition education in rural and poorer communities of South Africa in order to halt the escalating MetS epidemic as shown by a number of studies in the country [55–57].

## Limitations of the study

Adjusting for potential confounders boosted the strength of the study results. Face-to-face 24-h dietary recall interviews administered by trained interviewers on one weekday and on one weekend day decreased the underestimation possibility. However, 24-h dietary recall data for a 2-day period is not adequate because dietary habits typically differ each day from those on other weekdays. Thus our 24-h dietary recall data might have underestimated some nutrients as well as energy intake. Despite the importance of the outcomes of this study, authors also acknowledge the study limitations such as the cross-sectional design; we therefore cannot infer causality. Not all risk factors for MetS and dietary intake were included in the current study. As such, the current study focussed only on macronutrients. We recommend that future studies examine the micronutrient intake as this might show the utility. Considering of the socio-economic status (SES) and physical activity could have strengthened the findings. Therefore, future studies should focus on the combination of both macronutrient and micronutrient intake, SES and physical activity on the influence of MetS risk factors.

## Conclusion

MetS already appears to be entrenched in the rural communities in South Africa. Females seem to be the most vulnerable population group. In fact, the WC and HDL-C levels seem to be the main risk factors that explain the vulnerability of females to MetS compared to males. Males in the current study showed vulnerability to elevation of BP. The association between the dietary intake and MetS risk factors is a useful tool that will inform targeted interventions that may be useful in halting and eradicating MetS in a country that is undergoing a rapid nutrition transition. Moreover, identifying the groups that are at an increased risk and those that are in their early stages of MetS will help improve and prevent the increase of the MetS in future [58].
